# Entropy Optimization, Generalized Logarithms, and Duality Relations

**DOI:** 10.3390/e24121723

**Published:** 2022-11-25

**Authors:** Angel R. Plastino, Constantino Tsallis, Roseli S. Wedemann, Hans J. Haubold

**Affiliations:** 1 CeBio y Departamento de Ciencias Básicas, Universidad Nacional del Noroeste de la Província de Buenos Aires, UNNOBA, CONICET, Roque Saenz Peña 456, Junin B6000, Argentina; 2 Centro Brasileiro de Pesquisas Físicas and National Institute of Science and Technology for Complex Systems, Rua Xavier Sigaud 150, Rio de Janeiro 22290-180, RJ, Brazil; 3 Santa Fe Institute, 1399 Hyde Park Road, Santa Fe, NM 87501, USA; 4 Complexity Science Hub Vienna, Josefstädter Straße 39, 1080 Vienna, Austria; 5 Instituto de Matemática e Estatística, Universidade do Estado do Rio de Janeiro, Rua São Francisco Xavier 524, Rio de Janeiro 20550-900, RJ, Brazil; 6 Office for Outer Space Affairs, United Nations, Vienna International Center, 1400 Vienna, Austria

**Keywords:** generalized entropies, generalized logarithms, duality relations, entropy optimization, *S_q_* entropies, 05.90.+m

## Abstract

Several generalizations or extensions of the Boltzmann–Gibbs thermostatistics, based on non-standard entropies, have been the focus of considerable research activity in recent years. Among these, the power-law, non-additive entropies $S_{q}\equiv k\frac{1-\sum_{i}p_{i}^{q}}{q-1}\;\left(q\in R;\;S_{1}=S_{BG}\equiv -k\sum_{i}p_{i}\ln p_{i}\right)$ have harvested the largest number of successful applications. The specific structural features of the $S_{q}$ thermostatistics, therefore, are worthy of close scrutiny. In the present work, we analyze one of these features, according to which the *q*-logarithm function $\ln_{q}x\equiv \frac{x^{1-q}-1}{1-q}\;\left(\ln_{1}x=\ln x\right)$ associated with the $S_{q}$ entropy is linked, via a duality relation, to the *q*-exponential function characterizing the maximum-entropy probability distributions. We enquire into which entropic functionals lead to this or similar structures, and investigate the corresponding duality relations.

## 1. Introduction

Extensions of the maximum entropy principle based on non-standard entropic functionals [1,2,3,4] have proven to be useful for the study of diverse problems in physics and elsewhere, particularly in connection with complex systems [5,6]. These lines of enquiry were greatly stimulated by research into a generalized thermostatistics advanced in the late 80s, in which the canonical probability distributions optimize the $S_{q}$ power-law, non-additive entropies [7]. The $S_{q}$ thermostatistics was successfully applied to the analysis of a wide range of systems and processes in physics, astronomy, biology, economics, and other fields [8,9,10,11]. Motivated by the work on the $S_{q}$ entropies, researchers also explored the properties and possible applications of several other entropic measures, such as those introduced by Borges and Roditi [12], by Anteneodo and Plastino [13], by Kaniadakis [14], and by Obregón [15]. Recent reviews on these and other entropic forms can be found in [16,17]. These developments, in turn, led to the investigation of general properties of entropic variational principles, in order to elucidate which features are shared by large families of entropic forms, or are even universal, and, on the contrary, which features characterize specific entropies, such as the $S_{q}$ ones. Several aspects of general entropic variational principles have been studied along those lines, including, for instance, the Legendre transform structure [18,19,20], the maximum entropy–minimum norm approach to inverse problems [21], the implementation of dynamical thermostatting schemes [22,23], the interpretation of superstatistics in terms of entropic variational prescriptions [24], and the derivation of generalized maximum-entropy phase-space densities from Liouville dynamics [25].

Of all the thermostatistics associated with generalized entropic forms, the thermostatistics derived form the $S_{q}$ entropies has been the most intensively studied and fruitfully applied one. The $S_{q}$-thermostatistics exhibits some intriguing structural similarities with the standard Boltzmann–Gibbs one. The aim of the present contribution is to explore one of these similarities, within the context of thermostatistical formalisms based on general entropic functionals. As is well known, the Boltzmann–Gibbs entropy $S_{\mathrm{BG}}$ of a normalized probability distribution can be expressed as minus the mean value of the logarithms of the probabilities. Or, alternatively, as the mean value of the logarithms of the inverse probabilities. On the other hand, the probability distribution that optimizes $S_{\mathrm{BG}}$ under the constraints imposed by normalization and by the energy mean value, has an exponential form, where the exponential is the inverse function of the above mentioned logarithm function. In a nutshell: the entropy is the mean value of a logarithm (evaluated on the inverse probabilities), while the maximum-entropy probabilities are given by an exponential function, which is the inverse function of the logarithm. This structure turns out to be nontrivial, and, up to a duality condition, is shared by the $S_{q}$-thermostatistics. Indeed, it is possible to define a *q*-logarithm function, and its inverse function, a *q*-exponential, both parameterized by the parameter *q*, such that the $S_{q}$ entropy is the mean value of a *q*-logarithm (evaluated on the inverse probabilities), while the probability distribution optimizing $S_{q}$ has a *q*-exponential form. The alluded duality condition, however, imposes that the value of the *q*-parameter associated with the aforementioned *q*-logarithm should not be the same as the value of the parameter associated with the *q*-exponential. Both *q*-values are connected via the duality relation q↔2−q, which is ubiquitous in the $S_{q}$-thermostatistics. In the present work, we shall explore which entropic measures generate similar structures, linking the entropic functional, regarded as the mean value of a generalized logarithm, with the form of the maximum-entropy distributions.

This paper is organized in the following way. In Section 2, we provide a brief review of the $S_{q}$-thermostatistical formalism, focusing on the *q*-logarithm duality relation. In Section 3, we explore which entropic functionals give rise to structures, and duality relations, similar to those characterizing the $S_{q}$-thermostatistics. More general scenarios are considered in Section 3. Finally, some conclusions are drawn in Section 4.

## 2. $S_{q}$ Entropies, *q*-Logarithms, and *q*-Exponential Maximum-Entropy Probability Distributions

The $S_{q}$-thermostatistics is constructed on the basis of the non-additive, power-law entropy $S_{q}$ [5] defined as

(1) $$S_{q}\;=\;\frac{k}{1-q}\sum_{i=1}^{W}\left(p_{i}^{q}-p_{i}\right),$$

where q∈R is a parameter characterizing the degree of non-additivity exhibited by the entropy, *k* is a constant chosen once and for ever, determining the dimensions and the units in which the entropy is measured, and $\left\{p_{i},\;\;i=1,\ldots ,W\right\}$ is an appropriately normalized probability distribution for a system admitting *W* microstates. In what follows, we shall assume that k=1. The limit q→1 corresponds to the standard Boltzmann–Gibbs (BG) entropy, that is, $S_{1}=S_{\mathrm{BG}}=-k\sum_{i=1}^{W}p_{i}\ln p_{i}$. The power-law entropy $S_{q}$ constitutes a distinguished and founding member of the club of generalized entropies, which is nowadays the focus of intensive research activity [3,4,16].

The *q*-logarithm function, given by

(2) $$\ln_{q}\left(x\right)\;=\;\frac{x^{1-q}-1}{1-q}\;\;\left(x>0;\;\ln_{1}x=\ln x\right),$$

and its inverse function, the *q*-exponential

(3) $$\exp_{q}\left(x\right)=\left\{\begin{array}{cc} {\left[1+\left(1-q\right)x\right]}^{\frac{1}{1-q}}\;, & \mathrm{if}\;1+(1-q)x>0\;,\\ 0\;, & \mathrm{if}\;1+(1-q)x\leq 0\; \end{array}\right.$$

constitute essential ingredients of the $S_{q}$ thermostatistical formalism. For the sake of completeness, it is worth mentioning that sometimes people use an alternative notation for the *q*-exponential, given by $\exp_{q}\left(x\right)={\left[1+\left(1-q\right)x\right]}_{+}^{\frac{1}{1-q}}$. The *q*-logarithm and the *q*-exponential functions arise naturally when one considers the constrained optimization of the entropy $S_{q}$ [5,9]. Moreover, it is central to the *q*-thermostatistical theory that the $S_{q}$ entropy itself can be expressed in terms of *q*-logarithms,

(4) $$S_{q}\;=\;k\sum_{i=1}^{W}p_{i}\ln_{q}\left(\frac{1}{p_{i}}\right)\;=\;k\;\langle \ln_{q}\left(\frac{1}{p_{i}}\right)\rangle .$$

Note that, for q→1, the above equation reduces to the well-known one, $S_{\mathrm{BG}}=k\sum_{i=1}^{W}p_{i}\ln\left(\frac{1}{p_{i}}\right)$.

The gist of the $S_{q}$ thermostatistics is centered on the optimization of $S_{q}$ under suitable constraints. The $S_{q}$ entropic variational problem can be formulated using standard linear constraints or nonlinear constraints based on escort probability distributions [26,27]. When working with more general entropic functionals, it is not well understood what are the appropriate escort mean values to be used, and few or no concrete applications of escort mean values to particular problems have been developed. Consequently, in order to investigate and clarify the distinguishing features of the $S_{q}$ formalism within the context of more general entropic formalisms, it is convenient to restrict our considerations to the optimization of the $S_{q}$ entropy under linear constraints. The main instance of the $S_{q}$ variational problem is the one yielding the generalized canonical probability distribution, which corresponds to the optimization of $S_{q}$ under the constraints corresponding to normalization,

(5) $$\sum_{i=1}^{W}\;p_{i}\;=\;1,$$

and to mean energy. We assume that the *i*th microstate of the system under consideration, which has probability $p_{i}$, has energy $\epsilon_{i}$. The mean energy is then

(6) $$E\;=\;\sum_{i=1}^{W}\;p_{i}\;\epsilon_{i}.$$

Introducing the Lagrange multipliers α and β, corresponding to the constraints of normalization (5) and the mean energy (6), the optimization of $S_{q}$ leads to the variational problem

(7) $$\delta \left[S_{q}\;-\;\alpha \left(\sum_{i=1}^{W}\;p_{i}\right)-\beta E\right]\;=\;0,$$

yielding

(8) $$p_{i}^{q-1}=\frac{1}{q}\;\left[1\;-\;\left(q-1\right)\left(\alpha +\beta \epsilon_{i}\right)\right].$$

For later comparison with thermostatistical formalisms based on general entropic forms, it will prove convenient to recast the above equation as

(9) $$p_{i}^{q-1}=\;1\;+\;\left(q-1\right)\left[A\;-\;B(\alpha +\beta \epsilon_{i})\right],$$

with A=−1/q and B=1/q. At first glance, it might seem cumbersome to introduce the parameters A and B, since, within the context of the $S_{q}$-thermostatistics, they are simple functions of the entropic parameter *q*. The new parameters, however, will prove essential when exploring the duality properties exhibited by thermostatitsical formalisms based on other generalized entropies, and when comparing those properties with the ones corresponding to the $S_{q}$ entropy. In those scenarios, the parameters A and B have other values, depending on the parameterized form of the relevant entropic functionals. Using the A and B parameters, the maximum $S_{q}$ entropy probability distribution can be expressed in terms of a *q*-exponential, as follows:

(10) $$p_{i}=\;\exp_{\tilde{q}}\left[A-B(\alpha +\beta \epsilon_{i})\right]\;=\;\ln_{\tilde{q}}^{\left(-1\right)}\left[A-B(\alpha +\beta \epsilon_{i})\right],$$

where

(11) $$\tilde{q}\;=\;2-q.$$

Comparing now the Equation (4) for the entropy, with the Equation (10) for the probabilities optimizing the entropy, we see that the $S_{q}$ entropy can be expressed in terms of a *q*-logarithm function, while the optimal probabilities are given by an inverse *q*-logarithm function (that is, by a *q*-exponential function). However, the value of the *q*-parameter that appears in the first *q*-logarithm, associated with the entropy, does not coincide with the one, denoted by $\tilde{q}$, that appears in the inverse *q*-logarithm defining the optimal probabilities. This pair of *q*-values satisfy the duality relation (11). It is important to emphasize that the duality relation (11) has the property

(12) $$\tilde{\tilde{q}}\;=\;q.$$

In other words, the dual of the dual of *q* is equal to *q* itself. Note also that, in the Boltzmann–Gibbs limit, q→1, the duality relation reduces to $\tilde{q}=q=1$. The Boltzmann–Gibbs thermostatistics, regarded as a particular member of the $S_{q}$-thermostatistical family, is self-dual. The duality relation (12) between the values of the *q*-parameters characterizing two *q*-logarithm functions, can be reformulated as a duality relation between the *q*-logarithms themselves. Indeed, one has that

(13) $$\ln_{\tilde{q}}\left(x\right)\;=\;-\ln_{q}\left(\frac{1}{x}\right).$$

For q→1, the self-dual condition $q=\tilde{q}=1$ is obtained, and the relation (13) reduces to the well-known property of the standard logarithm, ln(x)=−ln(1/x).

## 3. Generalized Entropies and Logarithms

Now, we shall consider a generic trace-form entropy $S_{G}$. It can always be written in the form

(14) $$S_{G}\;=\;\sum_{i=1}^{W}\;p_{i}\;\ln_{G}\left(\frac{1}{p_{i}}\right),$$

expressed in terms of an appropriate generalized logarithm function $\ln_{G}\left(x\right)$. The specific form of the generalized logarithmic function $\ln_{G}\left(x\right)$ depends on which particular thermostatistical formalism one is considering. For example, in the case of the $S_{q}$-based thermostatistics, $\ln_{G}\left(x\right)$ is given by the generalized logarithm $\ln_{q}\left(x\right)$. Note that the subindex “*G*” stands for “generalized", and it does not represent a numerical parameter. In order to lead to a sensible entropy, the function $\ln_{G}\left(x\right)$ has to be continuous and two-times differentiable, has to comply with $\left(x\ln_{G}\left(1/x\right)\right)>0$ for 0<x<1 and $\lim_{x\to 0}\left(x\ln_{G}\left(1/x\right)\right)=\lim_{x\to 1}\left(x\ln_{G}\left(1/x\right)\right)=0$, and has to satisfy the concavity requirement given by $\frac{d^{2}}{dx^{2}}\left[x\ln_{G}\left(\frac{1}{x}\right)\right]<0$.

One can optimize the entropic measure (14) under the constraints imposed by normalization (5) and by the energy mean value (6). The corresponding variational problem reads

(15) $$\delta \left[S_{G}\;-\;\alpha \left(\sum_{i=1}^{W}\;p_{i}\right)-\beta E\right]\;=\;0,$$

where α and β are the Lagrange multipliers corresponding to the normalization and the mean energy constraints. The solution to the variational problem is given by a probability distribution complying with the equations

(16) $$\frac{1}{p_{i}}\ln_{G}^{\prime }\left(\frac{1}{p_{i}}\right)-\ln_{G}\left(\frac{1}{p_{i}}\right)=-\alpha -\beta \epsilon_{i},\;\;\;\left(i=1,\ldots ,W\right),$$

where $\ln_{G}^{\prime }\left(x\right)=\frac{d}{dx}\ln_{G}\left(x\right)$.

Equation (16) arises from a generic entropy optimization problem. Basically, the optimization of any trace form entropy leads to an equation of the form (16). Here, we want to consider a particular family of entropies, leading to maximum entropy distributions satisfying a special symmetry requirement. We want the maximum entropy distribution $p_{i}$ to be of the form

(17) $$p_{i}\;=\;\ln_{\tilde{G}}^{\left(-1\right)}\left(\xi_{i}\right),$$

where $\xi_{i}=A+B\left(-\alpha -\beta \epsilon_{i}\right)$, with A and B appropriate constants (B>0), and $\ln_{\tilde{G}}^{\left(-1\right)}$ is the inverse of a generalized logarithmic function $\ln_{\tilde{G}}\left(x\right)$, related to $\ln_{G}\left(x\right)$ through a *duality relationship*. A few clarifying remarks are now in order. First, $\xi_{i}$ is, up to the additive and multiplicative constants A and B, equal to the right-hand side of (16). Second, the constants *A* and *B* depend only on the form of the entropy (14), and not on any details of the system under consideration, such as the number of microstates *W*, the values of the microstates’ energies $\epsilon_{i}$, or the values of the Lagrange multipliers α and β. Last, the duality relation connecting the functions $\ln_{G}\left(x\right)$ and $\ln_{\tilde{G}}\left(x\right)$ is such that the dual of the dual is equal to the original function, that is

(18) $$\ln_{\tilde{\tilde{G}}}\left(x\right)\;=\;\ln_{G}\left(x\right).$$

Combining Equations (16) and (17), one obtains

(19) $$\frac{1}{p_{i}}\ln_{G}^{\prime }\left(\frac{1}{p_{i}}\right)-\ln_{G}\left(\frac{1}{p_{i}}\right)=\frac{1}{B}\left(\ln_{\tilde{G}}\left(p_{i}\right)-A\right).$$

Introducing the constants A=−A/B and B=1/B, the above equation can be cast in the more convenient form

(20) $$\frac{1}{p_{i}}\ln_{G}^{\prime }\left(\frac{1}{p_{i}}\right)-\ln_{G}\left(\frac{1}{p_{i}}\right)=A+B\ln_{\tilde{G}}\left(p_{i}\right).$$

For a given duality relation $\ln_{G}\left(x\right)\;\to \;\ln_{\tilde{G}}\left(x\right)$, and given values of the parameters *A* and *B*, Equation (20) can be regarded as a differential equation that has to be obeyed by the generalized logarithmic function $\ln_{G}\left(x\right)$. For solving the differential equation, one needs an initial condition, given by the value $\ln_{G}\left(x_{0}\right)$ adopted by the generalized logarithm at some particular point $x_{0}$. We shall assume, as an initial condition, that $\ln_{G}\left(1\right)=0$.

Different forms of the duality relation $\ln_{G}\left(x\right)\;\to \;\ln_{\tilde{G}}\left(x\right)$ are compatible with different forms of the generalized logarithm, and with different forms of the generalized entropy. In what follows, we shall explore some instances of duality relations, in order to determine which entropic forms are compatible with them.

### 3.1. The Duality Condition Satisfied by the $S_{q}$ Thermostatistics

Motivated by the $S_{q}$-based thermostatistics, we shall first adopt the duality condition

(21) $$\ln_{\tilde{G}}\left(x\right)\;=\;-\ln_{G}\left(1/x\right),$$

which is precisely the relation (13) satisfied by the $S_{q}$-thermostatistics. Equation (20) then becomes

(22) $$\frac{1}{p_{i}}\ln_{G}^{\prime }\left(\frac{1}{p_{i}}\right)-\ln_{G}\left(\frac{1}{p_{i}}\right)=A-B\ln_{G}\left(\frac{1}{p_{i}}\right).$$

Therefore, in order to find the form of $\ln_{G}\left(x\right)$, we have to solve the differential equation

(23) $$\ln_{G}^{\prime }\left(x\right)\;=\;\frac{1}{x}\;\left[A+\left(1-B\right)\ln_{G}\left(x\right)\right],$$

with the initial condition $\ln_{G}\left(1\right)=0$. The (unique) solution of Equation (23) is then

(24) $$\ln_{G}\left(x\right)\;=\;A\left(\frac{x^{1-B}-1}{1-B}\right).$$

We see that, up to the multiplicative constant *A*, the only generalized logarithmic function leading to an entropy optimization scheme compatible with the duality condition (21) is the *q*-logarithm

(25) $$\ln_{q}\left(x\right)\;=\;\frac{x^{1-q}-1}{1-q}.$$

The parameter *B* appearing in (22) coincides with the parameter *q* of the $S_{q}$-thermostatistics.

### 3.2. The Simplest Duality Relation

We shall now consider the simplest possible duality relation, which is

(26) $$\ln_{\tilde{G}}\left(x\right)\;=\;\ln_{G}\left(x\right).$$

In spite of its simplicity, this duality relation is worthy of consideration, because *it includes the standard logarithm (and the corresponding Boltzmann–Gibbs scenario) as a particular case*. It is interesting, therefore, to explore which entropic forms are compatible with the simplest conceivable condition (26), even if this exploration is not a priori motivated by a generalized entropy of known physical relevance.

Combining the general Equation (20) with the duality relation (26), one obtains

(27) $$\frac{1}{p_{i}}\ln_{G}^{\prime }\left(\frac{1}{p_{i}}\right)-\ln_{G}\left(\frac{1}{p_{i}}\right)=A+B\ln_{G}\left(p_{i}\right).$$

Then, we have to solve the ordinary differential equation

(28) $$\frac{1}{x}\ln_{G}^{\prime }\left(\frac{1}{x}\right)-\ln_{G}\left(\frac{1}{x}\right)=A+B\ln_{G}\left(x\right),$$

or, equivalently,

(29) $$\frac{d\ln_{G}}{dx}=\frac{1}{x}\left[\ln_{G}\left(x\right)+A+B\ln_{G}\left(\frac{1}{x}\right)\right],$$

with the condition $\ln_{G}\left(1\right)=0$. At first sight, Equation (29) may look like a standard ordinary differential equation. It has, however, the peculiarity that in the right-hand side of (29), the unknown function $\ln_{G}$ is evaluated at two different values of its argument: *x* and 1/x. This situation is similar to the one that occurs, for instance, with differential equations describing dynamical systems with delay. In the case of (29), this difficulty can be removed by recasting the equation as a pair of coupled ordinary differential equations. Let us introduce the functions

(30) $$\begin{array}{ccc} F(x) & = & \ln_{G}\left(x\right),\\ G(x) & = & \ln_{G}\left(1/x\right). \end{array}$$

The differential Equation (28) can be reformulated as the two coupled differential equations

(31) $$\begin{array}{ccc} \frac{dF}{dx}\;\; & = & \;\frac{1}{x}\left[F(x)+B\;G(x)+A\right],\\ \frac{dG}{dx}\;\; & = & \;-\frac{1}{x}\left[G(x)+B\;F(x)+A\right], \end{array}$$

with the conditions F(1)=G(1)=0. To find a solution for (31), we propose the ansatz

(32) $$\begin{array}{ccc} F(x) & = & c_{1}x^{\gamma_{1}}+c_{2}x^{\gamma_{2}}+c_{3},\\ G(x) & = & c_{1}x^{-\gamma_{1}}+c_{2}x^{-\gamma_{2}}+c_{3}. \end{array}$$

If one inserts the ansatz (32) into the differential Equations (31), one can verify that (32) constitutes a solution, provided that

(33) $$\begin{array}{ccc} \gamma_{1} & = & -\gamma_{2}\geq 0,\\ c_{1}/c_{2} & = & -\frac{1}{B}\left(1+\sqrt{1-B^{2}}\right),\;\;\;\;\;\;\;\;0\leq B^{2}\leq 1,\\ c_{3} & = & -A/(1+B), \end{array}$$

and

(34) $$\gamma =\sqrt{1-B^{2}},$$

where $\gamma =\gamma_{1}=-\gamma_{2}$. It follows from (33) and (34) that 0≤γ≤1, and that

(35) $$c_{2}=-\sqrt{\frac{1-\gamma }{1+\gamma }}\;\;c_{1}.$$

The relations (33)–(35), together with the initial conditions F(1)=G(1)=0, lead to

(36) $$F\left(x\right)\;=\;\frac{A}{1+B}\;\left(\frac{\sqrt{1+\gamma }\;\;x^{\gamma }-\sqrt{1-\gamma }\;\;x^{-\gamma }}{\sqrt{1+\gamma }\;-\;\sqrt{1-\gamma }}\;-\;1\right),$$

and

(37) $$G\left(x\right)\;=\;\frac{A}{1+B}\;\left(\frac{\sqrt{1+\gamma }\;\;x^{-\gamma }-\sqrt{1-\gamma }\;\;x^{\gamma }}{\sqrt{1+\gamma }\;-\;\sqrt{1-\gamma }}\;-\;1\right).$$

The solution to the system of differential Equations (31) is completely determined by the conditions F(1)=G(1)=0. Therefore, given these conditions, and for 0≤B≤1, the solution (36) and (37) is unique. Now, the entropy $S_{\gamma }$ compatible with the duality relation (26) is $S_{\gamma }=\sum_{i}\;p_{i}\;\ln_{G}\left(\frac{1}{p_{i}}\right)$, with $\ln_{G}\left(x\right)=F\left(x\right)$. Therefore, for 0≤B≤1, one has

(38) $$S_{\gamma }\;=\;\frac{A}{1+B}\;\sum_{i}\;\left(\frac{\sqrt{1+\gamma }\;\;p_{i}^{1-\gamma }-\sqrt{1-\gamma }\;\;p_{i}^{1+\gamma }}{\sqrt{1+\gamma }\;-\;\sqrt{1-\gamma }}\;-\;p_{i}\right),$$

which, after some algebra, can be recast in the more convenient form

(39) $$S_{\gamma }\;=\;\frac{A}{2}\;\left(\frac{\sqrt{1+\gamma }\;+\;\sqrt{1-\gamma }}{1+\sqrt{1-\gamma^{2}}}\right)\;\sum_{i}\;\left[\sqrt{1+\gamma }\;\;\left(\frac{p_{i}^{1-\gamma }-p_{i}}{\gamma }\right)+\sqrt{1-\gamma }\;\;\left(\frac{p_{i}^{1+\gamma }-p_{i}}{-\gamma }\right)\right].$$

Introducing now the parameters q=1−γ, (0≤q≤1) and $q^{*}=1+\gamma =2-q,\;\;$ ($1\leq q^{*}\leq 2$), the entropy (39) can be expressed as a linear combination of two $S_{q}$ entropies,

(40) $$S_{\gamma }\;=\;K\;\left(\sqrt{q^{*}}\;S_{q}\;+\;\sqrt{q}\;S_{q^{*}}\right),$$

where

(41) $$K\;=\;\frac{A}{2}\;\left(\frac{\sqrt{q}\;+\;\sqrt{q^{*}}}{1+\sqrt{q\;q^{*}}}\right).$$

In the limit B→1, which corresponds to γ→0, q→1, and $q^{*}\to 1$, the generalized entropy (40) is, up to a multiplicative constant, equal to the Boltzmann–Gibbs entropy $S_{\mathrm{BG}}$.

### 3.3. More General Duality Relations

It is possible to consider duality relations more general than the ones discussed previously. One can consider scenarios where the relation between a generalized logarithm and its dual is defined in terms of a pair of functions $h_{1,2}\left(x\right)$, as

(42) $$\ln_{\tilde{G}}\left(x\right)\;=\;h_{1}\;\left(\ln_{G}\left(h_{2}\left(x\right)\right)\right),$$

where the functions $h_{1,2}\left(x\right)$ satisfy

(43) $$h_{1}\left(h_{1}\left(x\right)\right)=x,\;\;\;\;\mathrm{and}\;\;\;\;h_{2}\left(h_{2}\left(x\right)\right)=x.$$

For example, the duality relation associated with the $S_{q}$ entropy corresponds to $h_{1}\left(x\right)=-x$ and $h_{2}\left(x\right)=1/x$, while the duality relation associated with the entropy $S_{\gamma }$ corresponds to $h_{1}\left(x\right)=h_{2}\left(x\right)=x$.

Other duality relations can be constructed, for instance, in terms of the Moebius transformations

(44) $$M\left(x\right)\;=\;\frac{m_{1}x+m_{2}}{m_{3}x+m_{4}},$$

with $m_{1}m_{4}-m_{2}m_{3}\neq 0$. The inverse of (44) is

(45) $$M^{\left(-1\right)}\left(x\right)\;=\;\frac{m_{4}x-m_{2}}{-m_{3}x+m_{1}}.$$

Moebius transformations that are self-inverse (that is, transformations coinciding with their own inverse: $M\left(x\right)=M^{\left(-1\right)}\left(x\right)$) are candidates for the functions $h_{1,2}\left(x\right)$ from which possible duality relations for generalized logarithmic functions can be constructed. Examples of self-inverse Moebius transformations are those of the form

(46) $$M\left(x\right)\;=\;\frac{m_{1}x+m_{2}}{m_{3}x-m_{1}},$$

which have $m_{4}=-m_{1}$. Notice that, for $m_{1}\neq 0$, the above form of M(x) depends on only two parameters, as follows: $M\left(x\right)=\frac{x+(m_{2}/m_{1})}{(m_{3}/m_{1})x-1}$. Another self-inverse Moebius transformation, not included in the family (46), is the identity function, M(x)=x, corresponding to $m_{1}=m_{4}\neq 0$ and $m_{2}=m_{3}=0$ (see also [28]). The duality relations corresponding to the entropic measures $S_{q}$ and $S_{\gamma }$ are both constructed in terms of particular instances of Moebius transformations. The duality relation associated with the entropy $S_{q}$ is constructed with $h_{1}\left(x\right)=-x$ and $h_{2}\left(x\right)=1/x$, which are the self-inverse Moebious transformation corresponding, respectively, to $m_{1}=1$, $m_{4}=-1$, and $m_{2}=m_{3}=0$, and to $m_{1}=m_{4}=0$ and $m_{2}=m_{3}=1$. The duality relation for the entropy $S_{\gamma }$ is constructed with $h_{1}\left(x\right)=h_{2}\left(x\right)=x$, which correspond to $m_{1}=m_{4}=1$ and $m_{2}=m_{3}=0$.

A generalized logarithmic function $\ln_{G}\left(x\right)$ defining a trace-form entropy (14), for which the associated entropic optimization principle leads to the duality relation (42), must satisfy the differential equation

(47) $$\begin{array}{ccc} \frac{1}{x}\ln_{G}^{\prime }\left(\frac{1}{x}\right)-\ln_{G}\left(\frac{1}{x}\right) & = & A+B\ln_{\tilde{G}}\left(x\right)\\ & = & A+B\;h_{1}\;\left(\ln_{G}(h_{2}\left(x\right)\right)), \end{array}$$

with the condition $\ln_{G}\left(1\right)=0$. For expression (14) to represent a sensible (i.e., concave) entropy, the generalized logarithm satisfying (47) has to comply with the requirement

(48) $$\frac{d^{2}}{dx^{2}}\left[x\;\ln_{G}\left(\frac{1}{x}\right)\right]=-B\;\frac{d}{dx}\left[h_{1}\;\left(\ln_{G}(h_{2}\left(x\right)\right))\right]<0.$$

For duality relations more general than the two ones already analyzed by us in detail (corresponding to the entropies $S_{q}$ and $S_{\gamma }$), the associated differential Equation (47) has, presumably, to be treated numerically.

### 3.4. Duality Relations: The Inverse Problem

One can also consider the following inverse problem. Given a parameterized family of non-negative, monotonically increasing functions J(x;λ), depending on one or more parameters (that we collectively denote by λ), find out if the inverse function $J^{\left(-1\right)}\left(x;\lambda \right)$ is related to a generalized logarithmic function defining a sensible entropy (14), and satisfying a duality relation (42) defined in terms of appropriate functions $h_{1,2}\left(x\right)$. The problem is the following: for the inverse function $J^{\left(-1\right)}\left(x;\lambda \right)$, determine if suitable functions $h_{1,2}\left(x\right)$ exist, and identify them. We assume that the integral

(49) $$I\;=\;\int_{0}^{1}\;J^{\left(-1\right)}\left(x^{\prime };\lambda \right)\;dx^{\prime },$$

converges.

In order to formulate this inverse problem, we consider a thermostatistical formalism, based on a generalized entropy, which yields optimizing-entropy canonical probability distributions of the form

(50) $$p_{i}\;=\;J\left(\xi_{i};\lambda \right),$$

where $\xi_{i}=A+B\left(-\alpha -\beta \epsilon_{i}\right)$. In the latter expression, α and β are, as usual, the Lagrange multipliers associated with normalization of mean energy, and A and B are constants, possibly depending on the parameters λ characterizing the function J(x;λ).

The associated entropy $S_{J}$ can be expressed as

(51) $$S_{J}\;=\;\sum_{i}C\left(p_{i}\right),$$

where the function C(x) is defined as the integral

(52) $$C\left(x\right)\;=\;\int_{x}^{1}\;\left[J^{\left(-1\right)}\left(x^{\prime };\lambda \right)\;-\;I\right]\;dx^{\prime }.$$

The function C(x) satisfies the following properties,

(53) $$\begin{array}{ccc} C(x) & > & 0,\;\;\;\;\mathrm{for}\;\;\;\;0<x<1,\\ C(0) & = & C(1)\;=\;0,\\ dC/dx & = & I-J^{\left(-1\right)}\left(x;\lambda \right),\\ d^{2}C/dx^{2} & = & -dJ^{\left(-1\right)}\left(x;\lambda \right)/dx\;<\;0, \end{array}$$

which guarantee that $S_{J}$, defined by (51), is a sensible entropy. For J(x)=exp(x), one has $J^{\left(-1\right)}\left(x\right)=\ln\left(x\right)$, I=−1, C(x)=−xln(x), and $S_{J}$ coincides with the Boltzmann–Gibbs entropy. If we compare the expression (51) for $S_{J}$ with the expression (14) for a generalized entropy in terms of a generalized logarithm, we find that the generalized logarithm associated with $S_{J}$ is

(54) $$\ln_{G}^{\left(J\right)}\left(\frac{1}{x}\right)\;=\;\frac{1}{x}\int_{x}^{1}\;\left[J^{\left(-1\right)}\left(x^{\prime };\lambda \right)\;-\;I\right]\;dx^{\prime },$$

or, equivalently,

(55) $$\ln_{G}^{\left(J\right)}\left(x\right)\;=\;x\int_{x^{-1}}^{1}\;\left[J^{\left(-1\right)}\left(x^{\prime };\lambda \right)\;-\;I\right]\;dx^{\prime }.$$

On the other hand, if we compare the form (17) for a generalized canonical distribution, with the form (50) corresponding to the function $S_{J}$, we obtain

(56) $$\ln_{\tilde{G}}^{\left(J\right)}\left(x\right)\;=\;J^{\left(-1\right)}\left(x;\lambda \right).$$

The present inverse problem consists of determining what type of duality relation, if any, exists between the functions (55) and (56). It seems that this is a difficult problem, which has to be tackled in a case-by-case way. As an intriguing example of this inverse problem, we can consider the one posed by probability distributions related to the Mittag-Leffler function $E_{a,b}\left(x\right)$ (see [29] and references therein). The Mittag-Leffler function is given, for a general complex argument *z*, by the power series expansion

(57) $$E_{a,b}\left(z\right)=\sum_{k=0}^{\infty }\frac{z^{k}}{\Gamma (b+ak)}\;,\;\;\;a,b\in C,\;\;\;ℜ\left(a\right)>0,\;\;\;ℜ\left(b\right)>0,\;\;\;z\in C\;,$$

with $E_{a}\left(z\right)\equiv E_{a,1}\left(z\right)$. Notice that, in the literature [29], the two parameters *a* and *b* characterizing the Mittag-Leffler function are sometimes referred to as α and β.

**Figure 1:**
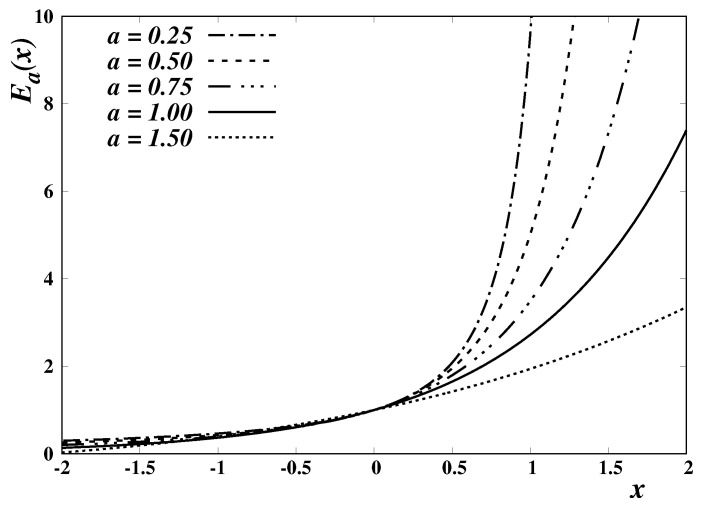
Plot of the Mittag-Leffler function $E_{a,b}\left(x\right)$, for b=1 and illustrative values of the parameter *a*; $E_{1,1}\left(x\right)=e^{x}$.

**Figure 2:**
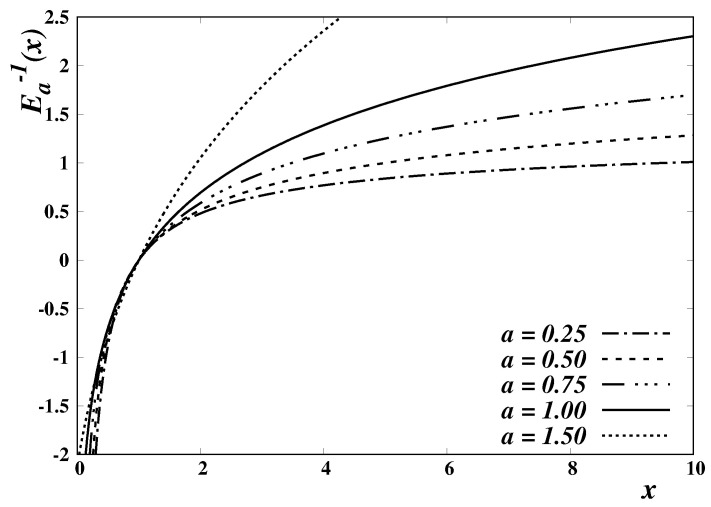
Plot of the inverse Mittag-Leffler function, $E_{a,b}^{\left(-1\right)}\left(x\right)$, for b=1 and specific values of the parameter *a*; $E_{1,1}^{\left(-1\right)}\left(x\right)=\ln x$.

The Mittag-Leffler function has several applications in physics and other fields. In particular, it plays a distinguished role in the study of non-standard diffusion processes involving fractional calculus operators [29]. In the present context, we consider only real values of the parameters (a,b) and real arguments. A few examples of the Mittag-Leffler function, and of its inverses, are respectively depicted in Figure 1 and Figure 2, for b=1 and different values of the parameter *a*.

**Figure 3:**
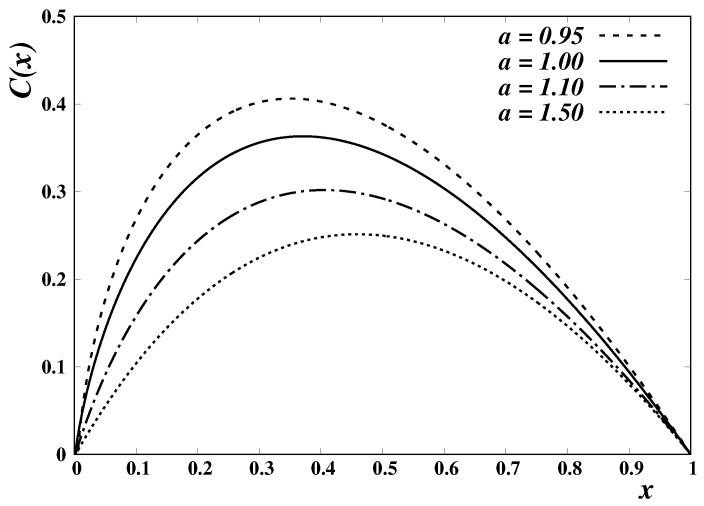
Plotof the function C(x) corresponding to $J\left(x\right)=E_{a}\left(x\right)$, for different values of the parameter *a*. The function C(x) appears in the definition of a trace-form entropic measure (51), and is given by Equation (52). For a=1, one has $E_{1}\left(x\right)=\exp\left(x\right)$ and C(x)=−xlnx.

In the context of a Mittag-Leffler-based thermostatistical formalism, some possible choices for the function J(x;λ) would be

(58) $$\begin{array}{ccc} J(x;\lambda )\; & = & \;E_{a,b}\left(x\right),\;\;\;\;\;\;\;\;\mathrm{or},\\ J(x;\lambda )\; & = & \;E_{a,b}\left(x^{2}\right), \end{array}$$

where λ=(a,b) is the set of parameters characterizing the Mittag-Leffler function. For each of these choices, provided that the values of the parameters λ are such that the appropriate conditions are fulfilled, it is possible to explore the existence of functions $h_{1,2}$ for which the Mittag-Leffler-related generalized logarithms, (55) and (56), satisfy a differential equation of the form (47). For $J\left(x;\lambda \right)=E_{a,1}\left(x\right)=E_{a}\left(x\right)$, the corresponding generalized entropy (51) is defined in terms of the function C(x), given by (52). A few examples of C(x), which we obtained by numerically solving the integrals (49) and (52) for particular values of the parameter *a*, are plotted in Figure 3.

## 4. Conclusions

Several generalizations or extensions of the notion of entropy have been advanced and enthusiastically investigated in recent years. The associated entropic optimization problems seem to provide valuable tools for the study of diverse problems in physics and other fields, particularly when applied to the analysis of complex systems. Among the growing number of entropic forms that have been advanced, the non-additive, power-law $S_{q}$ entropies exhibit the largest number of successful applications. It is clear by now, however, that the $S_{q}$ entropies are not universal: some systems or processes seem to be described by entropic forms not belonging to the $S_{q}$ family. Given this state of affairs, it is imperative to investigate in detail the properties of the various entropies, and of the associated thermostatistics, in order to elucidate and clarify the deep reasons that make them suitable for treating specific problems. In particular, the structural features of the $S_{q}$ thermostatistics are certainly worthy of close scrutiny. In the present work, we investigated one of these features, according to which the *q*-exponential function describing the maximum-entropy probability distributions are linked, via a duality relation, with the *q*-logarithm function in terms of which the $S_{q}$ entropy itself can be defined. We investigated which entropic functionals lead to this kind of structure and explored the corresponding duality relations.

The main take-home message of the present work is that there is a close connection between the aforementioned duality relations, and the forms of the entropic measures. The $S_{q}$ thermostatistics exhibits a particular duality connection, which, in the limit of the Boltzmann–Gibbs thermostatistics, reduces to a self-duality. We proved that there is no other entropic functional satisfying the duality relation associated with $S_{q}$, namely, Equation (21). This constitutes what may be regarded as a brand new uniqueness theorem leading to $S_{q}$, in addition to those already existing, such as the Enciso–Tempesta theorem [30] and those indicated therein. Assuming other types of duality relation, it is possible to formulate differential equations that lead to new entropic measures complying with the assumed duality. We studied in detail a duality relation leading to a differential equation that admits closed analytical solutions, and corresponds to a new generalized entropy, which we denoted by $S_{\gamma }$. The duality relations characterizing the entropies $S_{q}$ and $S_{\gamma }$ seem to be exceptional, in that the concomitant differential equations can be solved analytically. In many other cases, the differential equations resulting from duality relations have to be treated numerically. The investigation of these equations, associated with thermostatistical scenarios different from, or more general than, those based on the entropies $S_{q}$ and $S_{\gamma }$, would certainly be worthwhile. It would also be valuable to identify new duality relations admitting an analytical treatment. The exploration of the ensuing thermostatistical scenarios may suggest interesting new applications of generalized entropies. Another promising direction for future research is to extend the present study to scenarios involving non-trace-form entropies [31], or involving escort mean values [27,32]. We would be delighted to see further advances along these or related lines.
